# Transfers among Long-term Nursing Home Residents during the COVID-19 Pandemic

**DOI:** 10.1093/geroni/igab046.2709

**Published:** 2021-12-17

**Authors:** Ana Montoya, Chiang-Hua Chang, Pil Park, Julie Bynum

**Affiliations:** 1 University of Michigan, Ann Arbor, Michigan, United States; 2 University of Michigan, Ann ARbor, Michigan, United States

## Abstract

Transferring long-term nursing home residents between facilities can compromise the quality of life and be associated with functional decline, hospitalizations, and even death. This study aimed to examine transfer rates and identify risk factors associated with transfers among long-term nursing home residents before (2018-2019) and during the COVID-19 pandemic (2020). Using the Michigan state Minimum Data Set data 2018-2020, we identified long-term residents as those who stayed in nursing homes for at least 100 days each year (N=39,693, 39,454, and 35,575, respectively). We defined a facility-to-facility transfer as a direct transfer between two nursing homes. We first examined the likelihood of transfer by year using logistic regression models, adjusting for residents’ age, sex, race, and marital status. We then examined two health statuses that could be associated with a transfer: activities of daily living (ADL) and cognitive impairment. Finally, we compared transfers that occurred before COVID-19 (2018-2019) and during COVID-19 (2020), adjusting for residents’ demographic characteristics and health statuses. After adjustment, age was the only factor associated with transfers for all three years (Age>=80: AOR=0.61, 95% CI: 0.54-0.69; AOR=0.63, 95% CI: 0.55-0.72; AOR=0.71, 95% CI: 0.63-0.80, respectively). New risk factors in 2020 were Black race (AOR=1.22, 95% CI: 1.07-1.40) and requiring ADL assistance (AOR=1.24, 95% CI: 1.03-1.49). The COVID-19 period had higher transfer rate (unadjusted rates 2.9%, 2.7%, 3.5%, respectively) with 10% higher odds of transfer compared to before COVID-19 (AOR=1.10, 95% CI: 1.01-1.20). This finding suggests that COVID-19 has an impact on how nursing home transferred their long-term residents.

